# Supplementary material for: Clinical Impact of CDK4/6 Inhibitors in De Novo or PR− or Very Elderly Post-Menopausal ER+/HER2− Advanced Breast Cancers
Source: Cancers (Basel). 2023 Oct 26;15(21):5164. doi: 10.3390/cancers15215164 (PMC10647609; doi:10.3390/cancers15215164)
Supplement: Supplementary file 1 [file cancers-15-05164-s001.zip › cancers-2632998-supplementary.pdf]

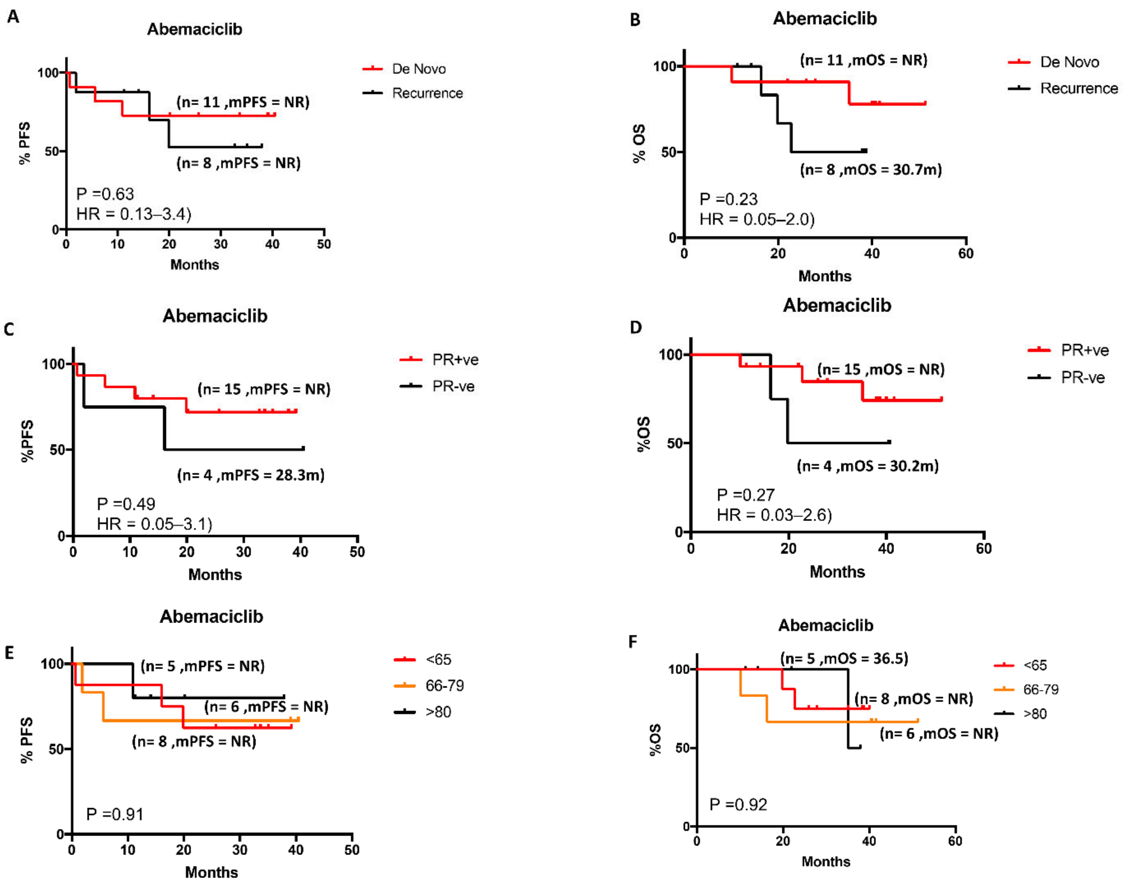

Figure S1. (A) Kaplan-Meier curve of progression free survival based on de novo versus recurrence in patients who received abemaciclib. (**B**) Kaplan-Meier curve of overall survival based on based on de novo versus recurrence in patients who received abemaciclib. **(C)** Kaplan-Meier curve of progression free survival based on based on PR expression status in patients who received abemaciclib. (**D**) Kaplan-Meier curve of overall survival based on based on PR expression status in patients who received abemaciclib. **(E)** Kaplan-Meier curve of progression free survival based on based on age in patients who received abemaciclib. (**F**) Kaplan-Meier curve of overall survival on age in patients who received abemaciclib.

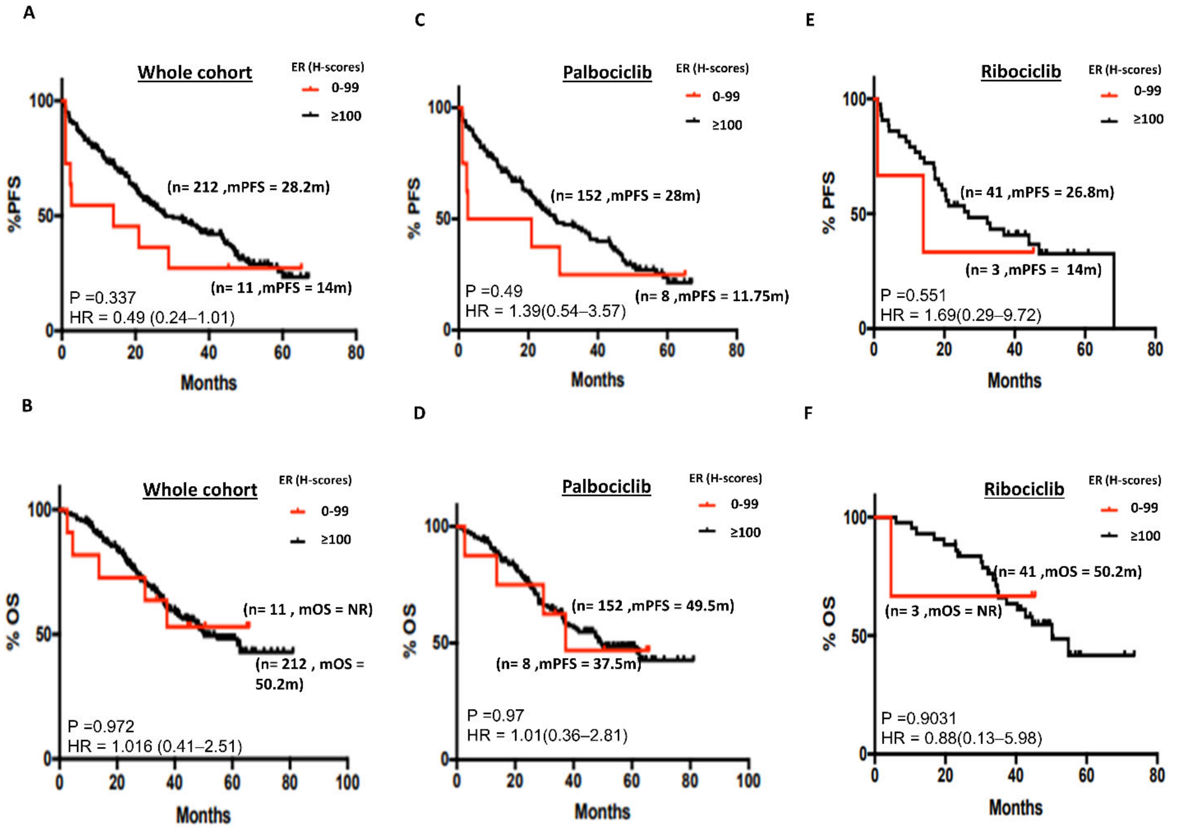

Figure S2. (A) Kaplan-Meier curve of progression free survival based on ER histochemical-score (0-99 versus ≥ 100) in the whole cohort. (**B**) Kaplan-Meier curve of overall survival based on ER histochemical-score (0-99 versus ≥ 100) in the whole cohort. **(C)** Kaplan-Meier curve of progression free survival based on ER histochemical-score (0-99 versus ≥ 100) in patients who received palbociclib. (**D**) Kaplan-Meier curve of overall survival based on ER histochemical-score (0-99 versus ≥ 100) in patients who received palbociclib. **(E)** Kaplan-Meier curve of progression free survival based on ER histochemical-score (0-99 versus ≥ 100) in patients who received ribociclib. (**F**) Kaplan-Meier curve of overall survival based on ER histochemical-score (0-99 versus ≥ 100) in patients who received ribociclib.

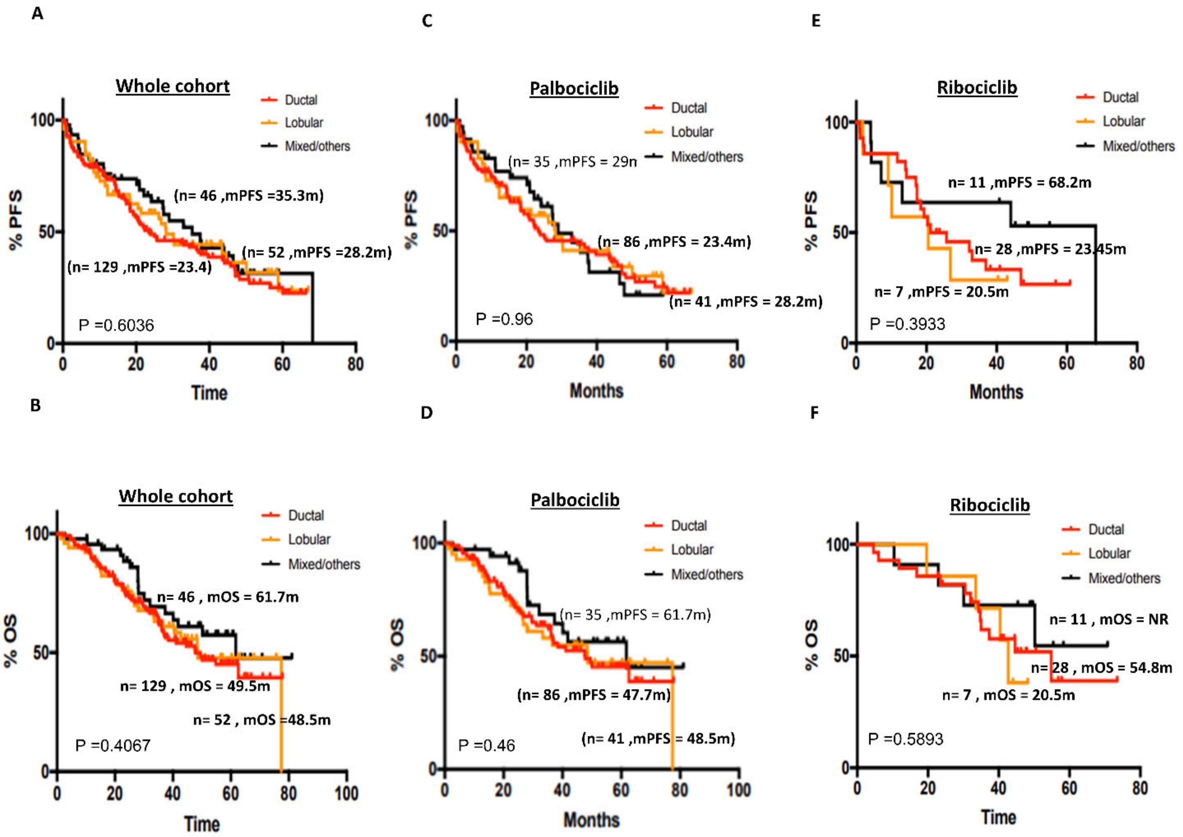

Figure S3. (A) Kaplan-Meier curve of progression free survival based on histopathology sub-type (ductal versus lobular versus mixed) in the whole cohort. (**B**) Kaplan-Meier curve of overall survival based on histopathology sub-type (ductal versus lobular versus mixed) in the whole cohort. **(C)** Kaplan-Meier curve of progression free survival based on histopathology sub-type (ductal versus lobular versus mixed) in patients who received palbociclib. (**D**) Kaplan-Meier curve of overall survival based on histopathology sub-type (ductal versus lobular versus mixed) in patients who received palbociclib. **(E)** Kaplan-Meier curve of progression free survival based on histopathology sub-type (ductal versus lobular versus mixed) in patients who received ribociclib. (**F**) Kaplan-Meier curve of overall survival based on histopathology sub-type (ductal versus lobular versus mixed) in patients who received ribociclib.

Supplementary Table S1. Overall survival and progression free survival percentage at five years

	Whole Cohort		Palbociclib Cohort		Ribociclib Cohort	
	5 year OS%	5 year PFS%	5 year OS%	5 year PFS%	5 year OS%	5 year PFS%
**Disease status**						
De Novo	65.84%	34.70%	62.38%	31.43%	82.50%	74.07%
Recurrence	36.91%	15.57%	37.98%	14.69%	27.98%	19.01%
**CDK4/6**i						
Palbociclib	48.54%	20.88%				
Ribociclib	42.33%	32.58%				
Abemaciclib	68.86%	66.80%				
**PR status**						
PR+	51.37%	26.73%	50.41%	22.66%	45.37%	41.82%
PR-	38.12%	20.27%	39.48%	21.07%	40%	13.33%
**Age**						
<65	52.00%	22.79%	55.27%	25.56%	36.86%	27.93%
66-79	51.16%	27.73%	50.17%	25.61%	49.36%	34.29%
>80	32.28%	11.89%	23.34%	0%	60%	60%
